# Identifying key genes in cancer networks using persistent homology

**DOI:** 10.1038/s41598-025-87265-4

**Published:** 2025-01-22

**Authors:** Rodrigo Henrique Ramos, Yago Augusto Bardelotte, Cynthia de Oliveira Lage Ferreira, Adenilso Simao

**Affiliations:** 1 University of São Paulo, ICMC, São Carlos, 13566-590 Brazil; 2 Federal Institute of São Paulo, São Carlos, 13565-820 Brazil

**Keywords:** Topological data analysis, Persistent homology, Cancer genomics, Driver genes, Pathways networks, Protein networks, Cancer, Cellular signalling networks, Network topology, Computer science

## Abstract

Identifying driver genes is crucial for understanding oncogenesis and developing targeted cancer therapies. Driver discovery methods using protein or pathway networks rely on traditional network science measures, focusing on nodes, edges, or community metrics. These methods can overlook the high-dimensional interactions that cancer genes have within cancer networks. This study presents a novel method using Persistent Homology to analyze the role of driver genes in higher-order structures within Cancer Consensus Networks derived from main cellular pathways. We integrate mutation data from six cancer types and three biological functions: DNA Repair, Chromatin Organization, and Programmed Cell Death. We systematically evaluated the impact of gene removal on topological voids ($\beta _2$ structures) within the Cancer Consensus Networks. Our results reveal that only known driver genes and cancer-associated genes influence these structures, while passenger genes do not. Although centrality measures alone proved insufficient to fully characterize impact genes, combining higher-order topological analysis with traditional network metrics can improve the precision of distinguishing between drivers and passengers. This work shows that cancer genes play an important role in higher-order structures, going beyond pairwise measures, and provides an approach to distinguish drivers and cancer-associated genes from passenger genes.

## Introduction

Cancer research has advanced significantly with the advent of high-throughput genomic data and the development of public databases. The availability of extensive genomic data has facilitated the development of computational and statistical methods in various fields, including the identification of cancer genes^1^. A major challenge in analysing mutation data lies in distinguishing between passenger and driver mutations. Passengers are the result of random genetic alterations or evolutionary processes and do not contribute to cancer development. In contrast, driver mutations are responsible for the onset and progression of the disease, making them targets for therapeutic intervention and personalised medicine^1,2^. In this work, in addition to drivers and passengers, we also use the term “cancer-associated genes” to refer to genes with publications associating them with cancer but are not present in driver databases.

Protein-protein interaction networks (PPIN) and pathway networks are graph-based models representing protein interactions within cells. PPIN encompasses the entire interactome, while pathway networks represent specific biological functions, working as subsets of the interactome^3^. Numerous computational approaches use the topology of PPIN and pathway networks to investigate cancer-related phenomena, such as mutual exclusivity, and to identify driver genes^4–7^.

Traditional network science measures mainly address individual nodes, communities, or the whole network. Although powerful, traditional methods can overlook the topological and structural significance of gene interactions between the node and community level. Given the limitations of traditional methods, the Persistent Homology (PH), a tool from algebraic topology, offers a novel way to analyse complex networks by capturing multi-dimensional features^8,9^. This approach enables the identification of higher-order structures in cancer networks, providing a deeper understanding of the roles that specific genes play in the context of these structures.

The objective of this study is to employ PH to identify genes that form higher-order structures within cancer networks derived from pathway networks and to explore their relationship with cancer. We constructed Cancer Consensus Networks (CCNs) using data from six types of cancer and three major biological functions: DNA Repair, Chromatin Organisation, and Programmed Cell Death. To evaluate the impact of each gene on topological voids ($\beta _2$ structures) within the CCNs, we systematically removed individual nodes and analysed the resulting changes. We then examine the role of these impactful genes in cancer.

Our findings reveal that every gene that affects $\beta _2$ structures is either a known driver or a cancer-associated gene, with the potential to be new drivers. The CCNs were constructed using mutated genes from various types of cancer. Given that most mutations are passengers^2,10^, we emphasise that removing passenger genes does not affect $\beta _2$ structures. Furthermore, we evaluated these impactful genes (known drivers or genes associated with cancer) using traditional network science measures, highlighting how centrality metrics alone are insufficient to fully characterise them. Not all known drivers or cancer-associated genes in the CCNs impact the formation of $\beta _2$ structures. However, no passenger gene has such an impact. Our method exhibits high precision with low to medium recall in distinguishing between drivers, cancer-associated genes, and passengers. Integrating higher-order topological features with traditional measures makes it possible to achieve a more comprehensive understanding of a gene’s role in cancer, which can be applied to evaluate candidate driver genes.

This work is organised as follows. The next two sections, “Cancer mutation data and reactome’s super pathways” and “Persistence homology”, present the theoretical background for developing this research. The “Methods” section details the data pipeline and our use of PH to characterise genes in CCNs. The “Results and discussion” section explores the removal of genes from networks, its impact on higher-order structures, and how drivers and cancer-associated genes play a critical role in it. Finally, we end our paper with the concluding remarks. A Supplementary material is also included, containing formal PH definitions, Python implementation, and associations of impacting genes with cancer pathways and antineoplastic drugs.

## Cancer mutation data and reactome’s super pathways

Advancements in DNA sequencing technologies have led to the generation of extensive genomic data. In the field of cancer research, databases such as the International Cancer Genome Consortium (ICGC) and the Cancer Genome Atlas (TCGA) offer datasets containing gene and mutation data for various types of cancer. Among the available datasets, the Mutation Annotation Format (MAF) is a commonly used tab-delimited file that connects patient samples, genes, and mutations. Each patient has one or more samples, each sample containing multiple genes linked to one or more mutations. The MAF file is frequently utilised in exploratory and computational approaches to identify driver genes and study patterns of mutual exclusivity^7,11^. In this work, we used cancer data from TCGA. Since TCGA deidentifies and anonymises all patient information, ethical approval was not required for this research.

Mutated genes in MAF files can be classified as either drivers or passengers. Drivers are genes whose mutations are causally linked to cancer^1^, with databases such as NCG^12^ and IntOGen^13^ offering lists of well-established drivers. These databases update their lists as new evidence emerges regarding a gene’s role in cancer. Passengers, on the other hand, are mutated genes present in the MAF file but are not relevant to cancer^1^. Distinguishing between drivers and passengers remains a critical challenge in cancer genomics^2^, leading to the development of numerous computational methods to identify new drivers^6^. In this paper, we consider the genes listed in these databases as “known drivers”, with high confidence in their role in cancer. All other mutated genes can be passengers or cancer-associated genes with the potential to be new drivers.

Pathways consist of sets of genes that collaborate to produce specific biological functions. As pathways are subsets of the entire PPIN, they are considerably smaller and provide meaningful information on the biological roles of their genes^3^. Recent research comparing human PPINs from various databases reveals substantial inconsistencies in their interactions and topological structures^14^. The same study shows that subnetworks, including pathway networks, are more consistent across different PPINs. These findings indicate that whole PPINs are incomplete and still evolving, with new interactions continuously being discovered, validated, or invalidated. In contrast, interactions within well-known pathways, such as those used in this study, are more established, making pathway networks a more reliable option compared to whole PPINs^14^.

The Reactome Knowledgebase (https://reactome.org) is an open access, peer-reviewed, expertly curated database focused on biological pathways^15^. It offers a variety of online bioinformatics tools designed for the analysis and visualisation of pathway-related data. Additionally, Reactome includes a PPIN derived from its pathway networks^16^. In 2020, Reactome introduced “Super Pathways”, a hierarchical organisation of pathways that begins with broad biological functions, such as Programmed Cell Death, and extends into more detailed subcategories, such as Apoptosis and Regulated Necrosis^17^. Reactome presents pathways as lists of genes, enabling the extraction of induced subgraphs from a PPIN to create Super Pathways Networks (SPNs), a procedure we explain in the “Methods” section.

## Persistence homology

Topological data analysis (TDA)^18–20^ is based on the principle that topology and geometry can be utilised to derive both qualitative and quantitative insights about the underlying structure of data. Topological methods rely on the definition of similarity or distance between data points, allowing comparisons between data sets that may exist in different coordinate systems.

**Figure 1:**
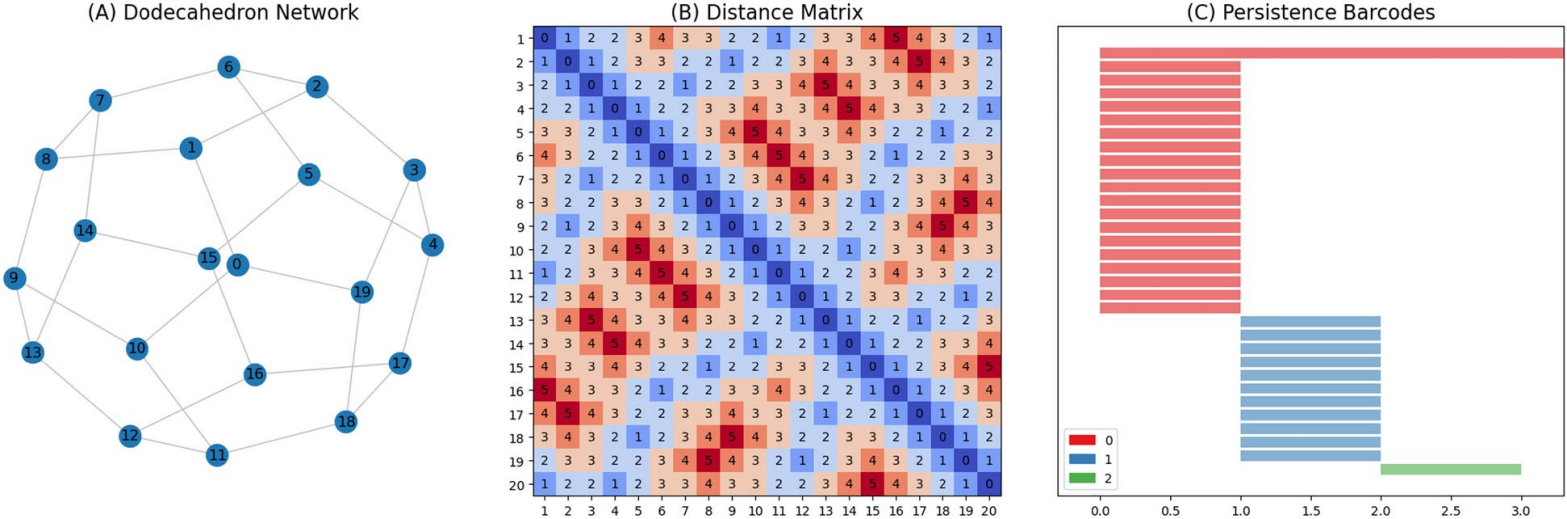
From network to persistence barcodes.

Persistent Homology (PH)^21^, a method within TDA, examines the topological features of data on various scales. PH identifies and quantifies the size and number of structures, such as connected components, cycles, and voids, by constructing a corresponding topological space from the data. The PH framework is built upon some fundamental concepts: simplicial complexes, filtrations, chains, and boundaries. Sections SM1 and SM2 with Figs. 1 and 2 in the Supplementary material formally define and illustrate these concepts. In this section, we provide an overview of PH and demonstrate its application in network analysis.

Typically PH is calculated over a point cloud, as exemplified in the supplementary material. However, PH can also be computed over a network by defining a metric space based on a distance matrix calculated by pairwise distances between nodes. Fig. 1 demonstrates this process. Fig. 1A shows a network that resembles a dodecahedron, with 20 nodes and 30 edges. Fig. 1B shows a × distance matrix calculated using the shortest path length between nodes. This matrix is the metric space used to calculate PH. Fig. 1C presents the Persistence Barcode, a plot normally used to visualise structures found during the PH. We will detail this in the next section.

### Persistence, barcodes and betti numbers

PH identifies the topological structures within the data. During the filtration step (explained in the Supplementary material), structures are born at a given time and die at another. Significant structures persist longer than noise structures and are meaningful for characterising the data. Persistence barcodes represent the birth and death of topological structures across multiple scales. In Fig. 1C, the bar colours represent different dimensions: red bars indicate connected components, blue bars indicate cycles (2-dimensional holes) and green bars indicate voids (3-dimensional holes). The X-axis of Fig. 1C shows the passage of time, i.e., the filtration process. Twenty red bars appear at time 0, and 19 persist until time 1, when the filtration process connects all loose connected components to one. This connection occurs at time 1 because the edges in Fig. 1A weight 1. At time 1, the dodecahedron faces are identified and persist for 1 tick of time. At time 2, a void is identified, representing the empty space inside the dodecahedron network. In summary, PH successfully identified the topological structures in Fig. 1A, and the persistence barcode is a way to represent them.

Betti numbers quantify the topological features of a space. Specifically, the *k*-th Betti number $\beta _k$ represents the number of *k*-dimensional holes in the data. $\beta _0$ counts the number of connected components, $\beta _1$ counts the number of cycles, and $\beta _2$ counts the number of voids. In Fig. 1C, we have $\beta _0 = 20$, $\beta _1 = 11$, and $\beta _2 = 1$. As a polyhedron, the dodecahedron consists of 12 pentagonal faces. However, persistence homology identified only 11 cycles because not all faces contribute to distinct cycles. The edges of the “missing” cycle are shared with adjacent cycles, thereby not forming an independent cycle. Betti numbers provide a convenient method for quantifying the structures represented in Persistence Barcodes. In this work, we focus on using Betti numbers rather than barcodes, as our primary concern is the number of structures in the network and the impact individual genes have on them.

### Persistence homology in cancer studies

PH is an innovative tool in data science and has made contributions in many fields, such as network science, physics, chemistry, biology, and medicine^22–27^, thanks to its ability to analyse high-dimension datasets and extract meaningful features from complex data.

In cancer studies, PH has been applied in various contexts, including image analysis, protein networks, gene expression networks, and point clouds. Specifically, PH has been used to evaluate prostate cancer in order to improve the Gleason grading system by capturing structure features independently of Gleason patterns. By computing topological representations of prostate cancer histopathology images, PH demonstrates the ability to group these images into unique groups through a ranked persistence vector. This method showed sensitivity to specific substructure groups within single Gleason patterns, offering a higher granularity than existing measures. The topological representations generated by PH could improve future approaches for better diagnosis and prognosis^28^.

Furthermore, PH has been utilised in the study of protein interactions in the KEGG database to inform cancer therapy by analysing the correlation between Betti numbers and patient survival^9^. In the context of gene expression networks, PH has been employed to examine gene interactions, uncovering structural features of the disease. It highlights significant deviations in the network topology between cancerous and healthy cells, emphasising the importance of cycles in cancer cells and voids in healthy cells^8^.

Moreover, PH has been applied in tumour segmentation of Hematoxylin and Eosin stained histology images to enhance computer-aided diagnosis systems. This approach segments tumours in whole-slide images by analysing the degree of connectivity among nuclei through persistent homology profiles, outperforming convolutional neural networks^29^. Lastly, PH has been used to characterise comparative genomic hybridisation profiles in breast cancer, providing a deeper understanding of chromosome amplifications and deletions in an individual’s genome. The results were aligned with previous studies and distinguished between cancer recurrence frequencies in chemotherapy-treated and nontreated patient populations, highlighting the potential of PH in genomic data analysis^30^.

## Methods

We selected three SPNs, Chromatin Organisation (CHR), DNA Repair (DNA), and Programmed Cell Death (PCD), due to the roles these biological processes play in cancer development^31–33^. Furthermore, these networks exhibit a high proportion of known driver genes^34^, making them suitable for our study. Although other SPNs, such as Gene Expression and Signal Transduction, are also relevant to cancer, their extensive size, comprising over a thousand nodes, renders them computationally infeasible for analysis using the Vietoris-Rips complex in PH analysis due to the prohibitive combinatorial costs involved.

**Figure 2:**
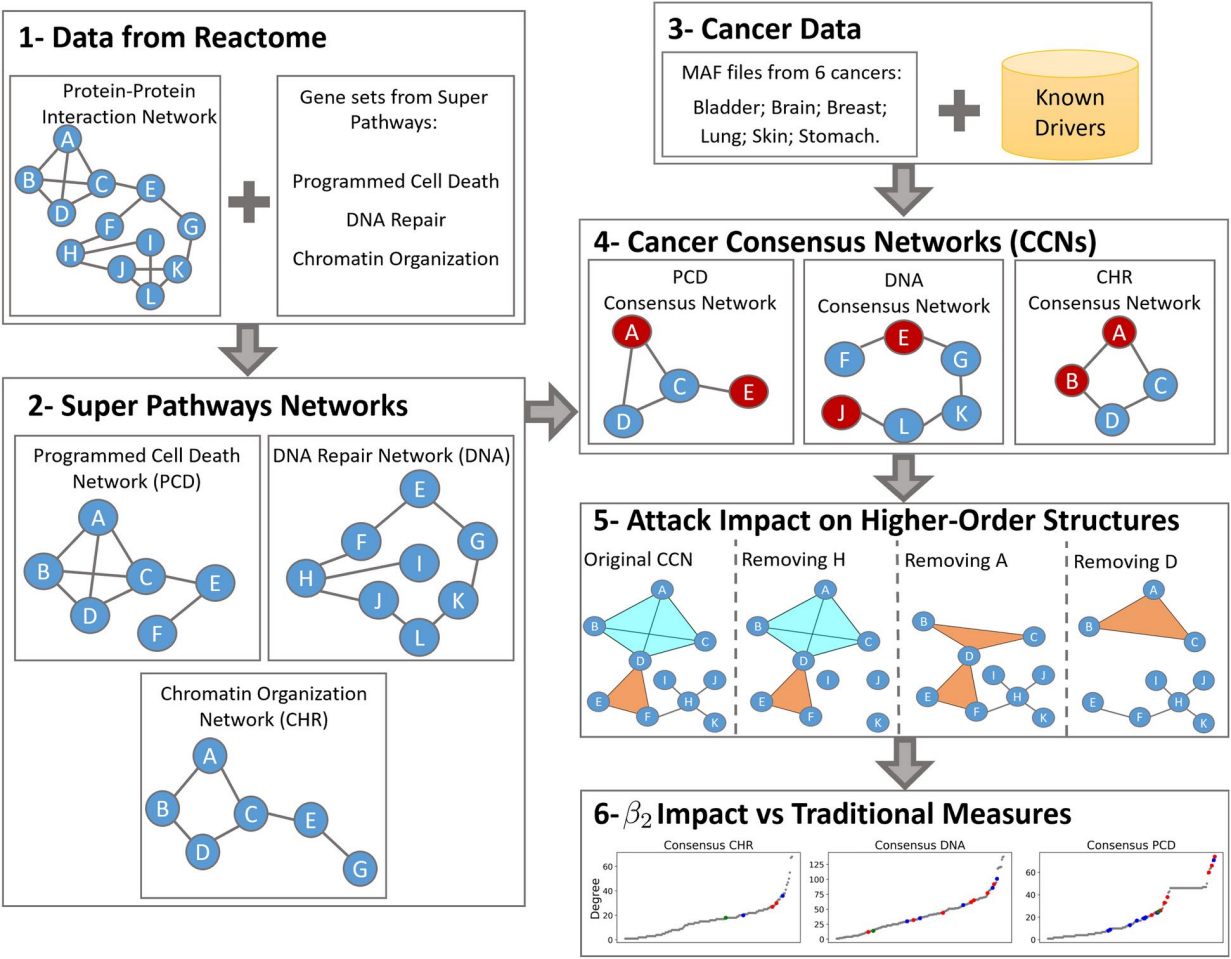
Data pipeline: We gather data from different databases to create Cancer Consensus Networks (CCNs), integrating data from three main biological functions with cancer-specific information. After that, we analyse the topological role of drivers and non-drivers in relation to their impact on higher-order structures.

The selected pathway networks represent the proteins and interactions present in normal and healthy cells. To associate these networks with cancer, we created the CCNs using mutation data from six types of cancer: Bladder, Breast, Head and Neck, Lung, Skin, and Stomach. Mutation data was obtained from MAF files in a TCGA pancancer study^35^. Figure 2 shows the pipeline used in this work, while algorithms further detail steps 3, 4, and 5.

In the first step, we collected data from the Reactome PPIN and Reactome pathways. In the second step, we adopted a method similar to our previous research^34^, where we generated SPNs by extracting induced subgraphs from the Reactome PPIN using gene sets linked to Super Pathways. The third phase was conducted independently of the previous steps. We selected genes that were mutated in at least four of the six MAF files corresponding to different types of cancer. Furthermore, we identified known driver genes by considering the combined data from the intOGen^13^ and NCG^12^ driver databases. Algorithm 1 detail the third step. The input *allGenes*, represent the genes present in all six MAF files.

**Algorithm 1:**
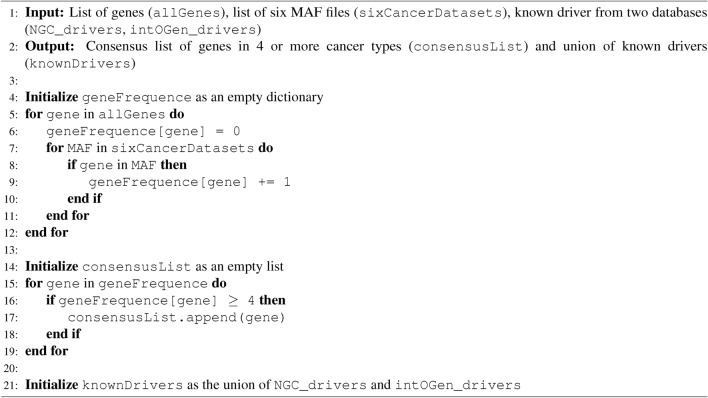
Consensus List Creation and Known Drivers Integration

Step four depends on steps two and three, since we use the *consensusList* from Algorithm 1 to extract induce subnetworks from each SPN, creating three CCNs. We also identify genes in the CCNs that are known drivers, represented in Fig. 2 as red nodes, using the *knownDrivers* from Algorithm 1. The original SPNs for CHR, DNA and PCD contain 221, 300, and 206 nodes, respectively. Their corresponding CCNs reduced the nodes to 162 (73%), 233 (78%), and 170 (83%). The number of driver genes in CHR, DNA, and PCD are 45, 46, and 26, respectively. In particular, the consensus networks retained at least 93% of the original driver genes. Although the total number of nodes in the consensus networks decreased by approximately 22% compared to the original SPNs, the reduction in driver genes was only 7%. Algorithm 2 corresponds to the fourth step and presents network manipulation functions from the Python library NetworkX^36^ at a high level of abstraction.

**Algorithm 2:**
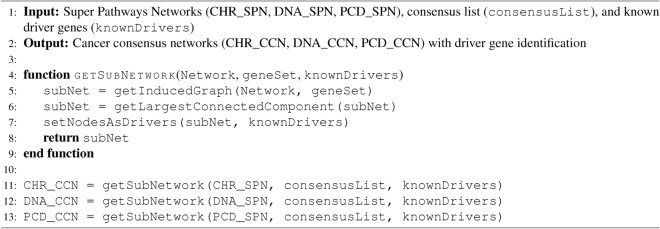
Creating CCNs from SPNs and Identifying Driver Genes

The fifth step in Figure 2 summarises the analysis we performed to characterise nodes regarding their topological role in higher-order structures. It begins by calculating the PH for each CCN and recording the $\beta _2$ value using the Vietoris-Rips complex^19^. In this work, we only focus on $\beta _2$ impact, since they are topologically more significant, are built using $\beta _1$, and their removal can increase the number of $\beta _1$. In the Fig. 2 example, the original CCN contains one cycle, formed by the nodes D, E, F, and one void, formed by the nodes A, B, C, D. Following this initial characterisation of the network, we systematically remove each node, one at a time, from the network and measure its impact on the $\beta _2$ value compared to the original CCN. In Fig. 2 example, removing node *H* creates three new connected components, but does not affect any higher-order structures. *H*’s impact can not be measured using PH, but can be measured by traditional network science measures, as previously done in the context of SPN and drivers^34^. On the other hand, removing node *A* barely affects the network by traditional measures, but it has a relevant impact on higher-order structures. Node *A* removal destroys a void ($\beta _2$) and creates a new cycle ($\beta _1$). Contrary to nodes *H* and *A*, node *D* significantly impacts both traditional measures and higher-order structures.

Algorithm 3 corresponds to the fifth step and presents PH calculations from the Python library GUDHI^37^ at a high level of abstraction. The supplementary material details the implementation of *fromNetworkToPH* and *getOnlyB2* in Python, where we also discuss parameters for the Vietoris-Rips filtration in the supplementary Figs. 3 and 4. The Algorithm 3 outputs are used in step 6 and in tables from the next section.

**Algorithm 3:**
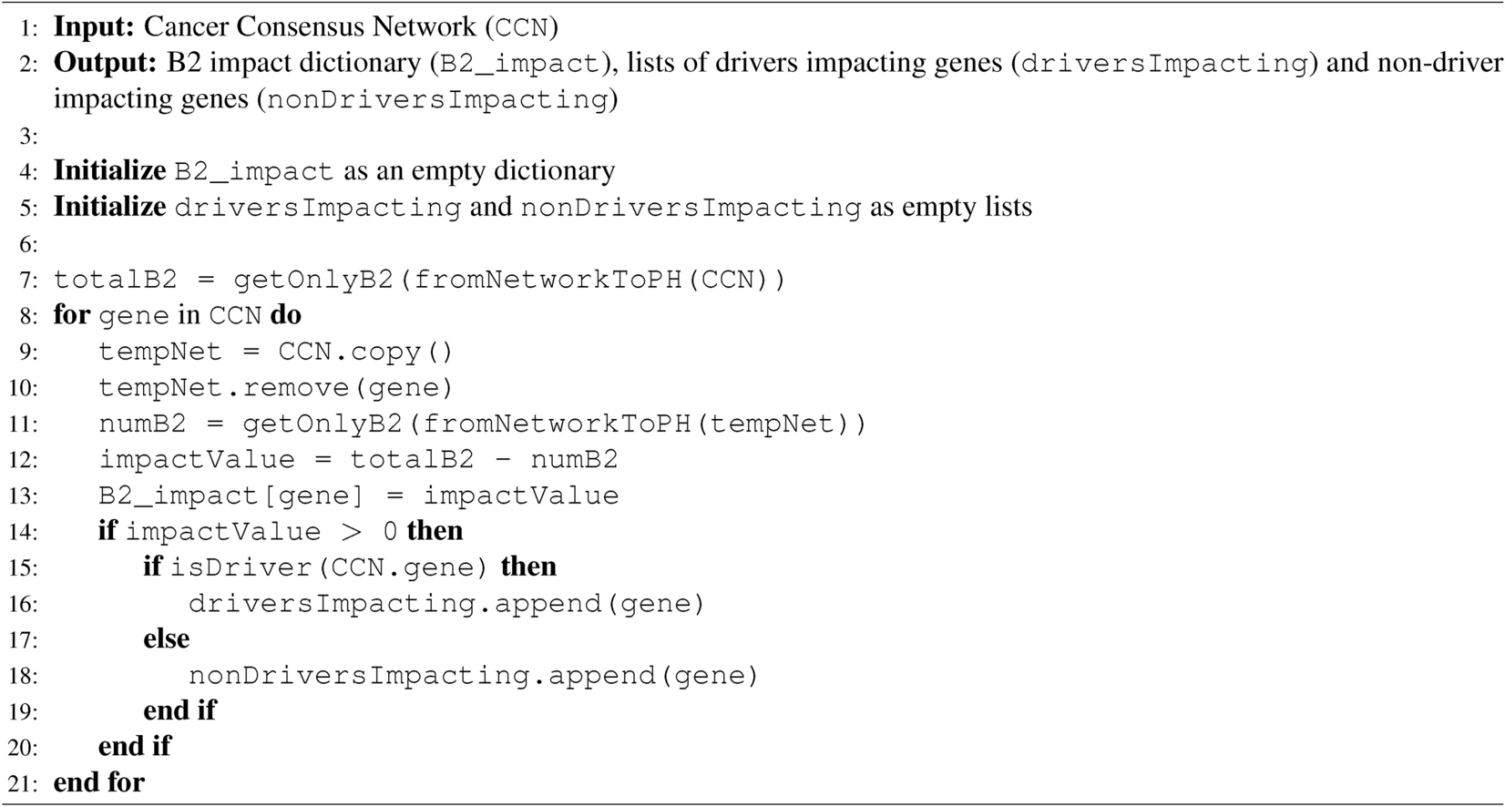
Calculating $\beta _2$ Impact for a CCN

The sixth and final step in Fig. 2 illustrates the second analysis performed to characterise the nodes. For each CCN, we calculate four centrality measures: degree, clustering, betweenness, and closeness. We then identify the position of the nodes that affected $\beta _2$ in the initial analysis. This step aims to compare the novel approach introduced in this paper, i.e., the impact of node on $\beta _2$, with traditional centrality measures.

## Result and discussion

The main objective of this work is to use PH to identify genes that form higher-order structures in CCNs and explore their relationship to cancer. By applying our proposed methodology, we assess the impact of each gene on the CCN’s $\beta _2$ by individually removing nodes. Our results demonstrate that every node impacting $\beta _2$ structures is either a known driver or a gene associated with cancer, which potentially represents new drivers. The CCNs are constructed using mutated genes from various types of cancer. Given that most mutations are passengers^2,10^, we emphasise that removing passengers does not affect $\beta _2$ structures. In addition, we analyse these impactful genes (known drivers or cancer-associated genes) using traditional network science measures and discuss how centrality measures alone fail to fully capture them. We also conduct an enrichment analysis of impactful genes and compare our approach with other methods that use high-order structures to study driver genes in PPINs.

### Impact on $\beta _2$ by single node removal

We calculated the PH for each CCN, identifying two $\beta _2$ structures in the CHR CCN, four $\beta _2$ structures in the DNA CCN, and ten $\beta _2$ structures in the PCD CCN. The PCD CCN, despite being the smallest network, exhibited the highest complexity in higher-order structures. Table 1 lists every gene that impacts $\beta _2$ structures in each CCN, highlighting in bold known drivers.

**Table 1:** Impact on $\beta _2$ structures by single node removal.

CCN	$\beta _2$ Impact	GENES
CHR	− 1	ACTL6A, BRMS1, **RELA**, **SMARCE1**, WDR77
DNA	− 1	**ATM**, **EP300**
DNA	− 2	**ABL1**, ACTL6A, **ATR**, **FANCD2**, **HERC2**, KAT5, PCNA, POLN, RAD51, **XPA**, XRCC6
PCD	− 1	**AKT1**, APAF1, BAD, BIRC2, CASP1, **CTNNB1**, MAPT, **RIPK1**, ROCK1, **STAT3**, **STUB1**, **TNFSF10**
PCD	− 2	**HSP90AA1N**, **PTK2**
PCD	− 3	**CASP3**, CASP6, **CASP8**
PCD	− 5	**TP53**

Bold names are known drivers.

CHR CCN is the least complex network, with five genes destroying one $\beta _2$ structure. In the DNA CCN, most impacting genes affected two $\beta _2$ structures. The PCD CCN, the most complex network, exhibited a different pattern, with the majority of impacting genes affecting only one $\beta _2$ structure. Five of the six genes that impacted more than one $\beta _2$ structure are known drivers. In particular, TP53, one of the most well-known genes in cancer research and frequently mutated across various types of cancer^38^, stands out for its ability to independently destroy five $\beta _2$ structures. Most of the known drivers in the analysed CCNs did not impact $\beta _2$ structures. We hypothesise that these genes may be involved in even higher-dimensional structures, beyond $\beta _2$. However, the exponential computational cost of performing Vietoris-Rips filtration restricts such an analysis. This limitation suggests an avenue for future research to develop a filtration method specific to cancer networks that could reduce computational costs and enable the exploration of these higher-dimensional structures.

**Figure 3:**
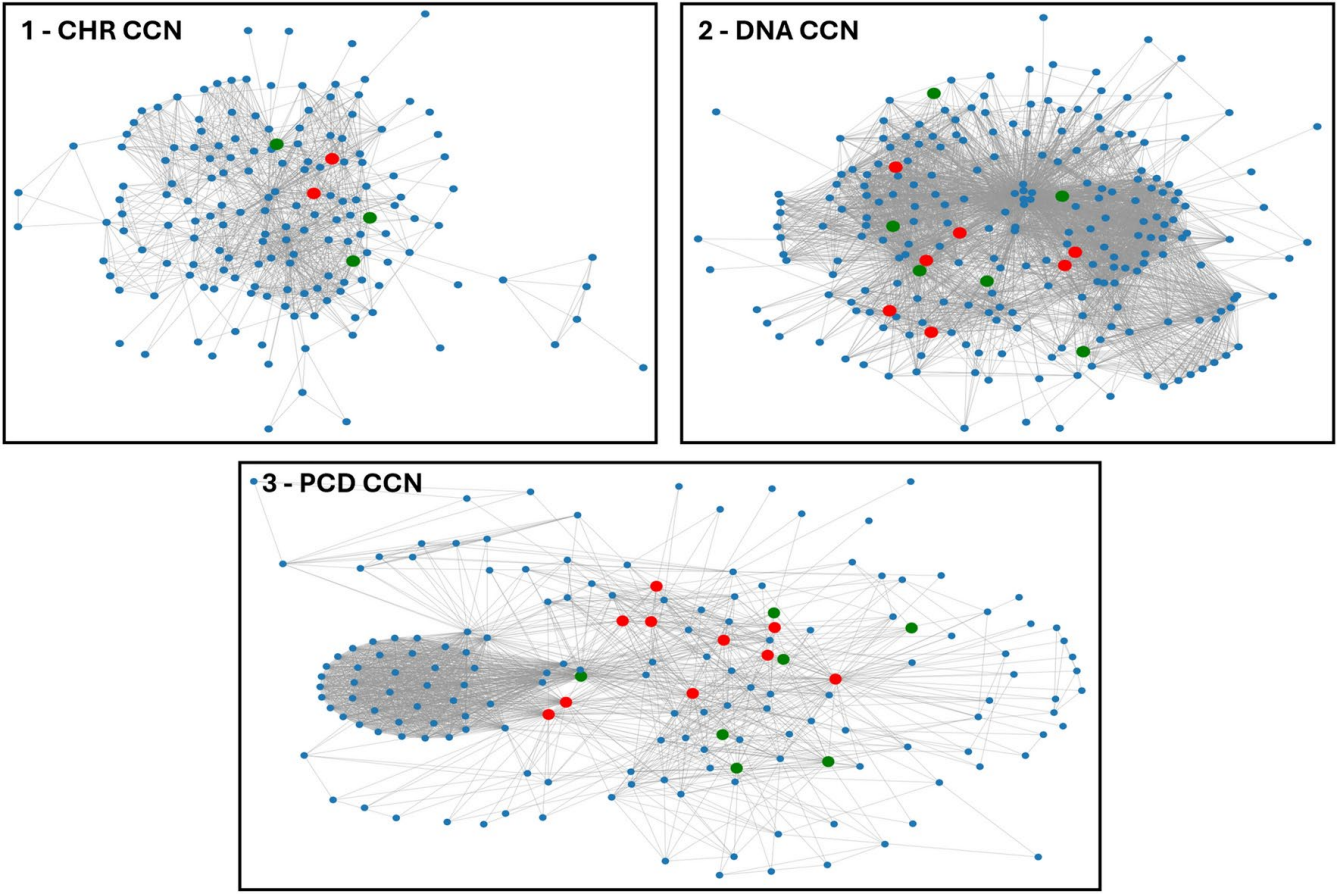
Visualization of CCNs. Red nodes are known drivers that impact $\beta _2$. Green nodes are cancer-associated genes that impact $\beta _2$. Blue nodes do not affect $\beta _2$.

Table 1 lists 35 unique genes, of which 20 are identified as known drivers according to the combined data from the NCG and IntOGen databases. Table 2, details these 35 impacting genes as we provide the most recent publications for genes not found in driver databases, and the most recent publications associating them with cancer. In particular, all 15 genes not found in drivers database are drug targets or related to cancer. Figure 3 shows the CCNs to provide insights into the network’s composition and the roles of impacting genes within it. In the figure, red nodes represent known driver genes that impact $\beta _2$, green nodes represent cancer-associated genes that impact $\beta _2$, and blue nodes represent genes that do not affect $\beta _2$.

**Table 2:** All 35 genes impacting $\beta _2$ structures in CCNs. 20 are known drivers listed in the NCG or IntOGen databases. The Literature column presents the most recent publication associating the remaining 15 genes with cancer.

Gene	NCG	IntOGen	Literature
ABL1	X	X	
ACTL6A	–	–	Association with squamous cell carcinoma^39^
AKT1	X	X	
APAF1	–	–	Melona drug target^40^
ATM	X	X	
ATR	X	X	
BAD	–	–	Association with triple-negative breast cancer^41^
BIRC2	–	–	Head and neck drug target^42^
BRMS1	–	–	Metastasis suppressor in breast cancer^43^
CASP1	–	–	Association with acute myeloid leukemia^44^
CASP3	X	–	
CASP6	–	–	Association with pancreatic cancer^45^
CASP8	X	X	
CTNNB1	X	X	
EP300	X	X	
FANCD2	X	X	
HERC2	X	–	
HSP90AA1	–	X	
KAT5	–	–	Association with hepatocellular carcinoma^46^
MAPT	–	–	Association in pan-cancer^47^
PCNA	–	–	Drug target in multiple cancers^48^
POLN	–	–	Association in nasopharyngeal carcinoma^49^
PTK2	X	–	
RAD51	–	–	Potential therapeutic target^50^
RELA	X	X	
RIPK1	X	X	
ROCK1	–	–	Association with cancreatic cancer^51^
SMARCE1	X	–	
STAT3	X	X	
STUB1	X	–	
TNFSF10	X	–	
TP53	X	X	
WDR77	–	–	Association with prostate cancer^52^
XPA	X	–	
XRCC6	–	–	Association with lung cancer chemotherapy^53^

The CCNs are extracted from SPNs using mutations from cancer patients, where the majority of mutations are passengers (i.e. not related to cancer). The results showed no $\beta _2$ impact upon removing passenger mutations, only consolidated known drivers or genes associated with cancer caused impact in higher-order structures.

### Impacting genes and centrality measures

Taking into account traditional network science measures, drivers are known to have a high degree and work as hubs^54^, while some drivers genes have small degree^34^. Other works indicate that drivers can be categorised using additional centrality measures^55,56^. When characterising cancer driver genes, one of the key challenges lies in identifying drivers in the long tail of distributions associated with measures from protein networks and mutation data^5^, as many methods are affected by “ascertainment bias”, which tends to favour frequently mutated genes and network hubs^57^. Here, we discuss whether genes impacting $\beta _2$ structures can be characterized using four centrality measures.

**Figure 4:**
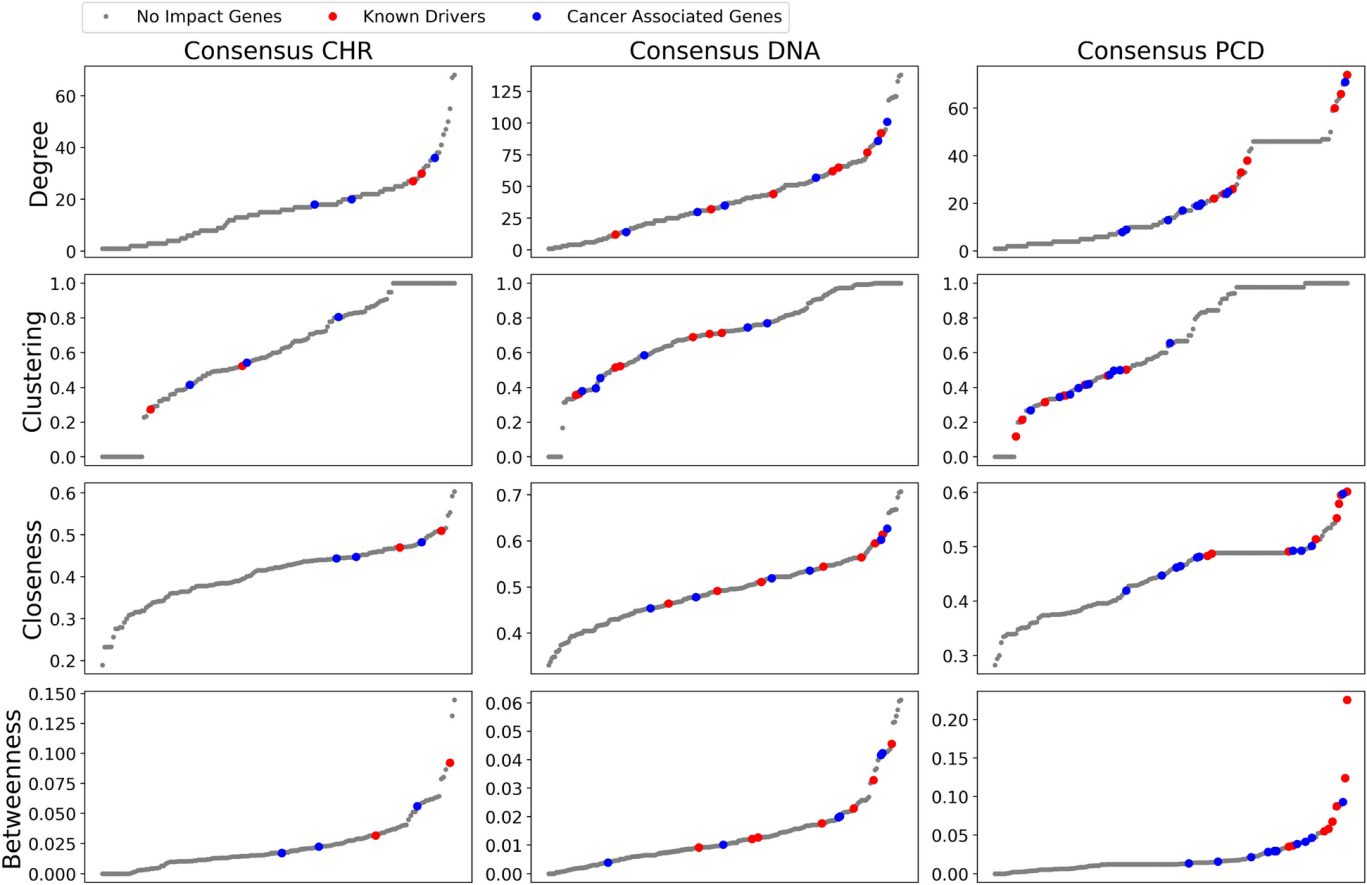
Centrality distributions for CCNs. Grey points represent genes whose removal does not affect $\beta _2$. Red and blue points indicate genes whose removal reduces $\beta _2$, with red points representing known drivers and blue points genes associated with cancer.

Figure 4 displays the distributions of four centrality measures for all genes within each CCN. Grey points represent genes whose removal does not impact $\beta _2$, while red and blue points indicate genes whose removal decreases $\beta _2$, which correspond to the genes listed in Tables 1 and 2. Red points are known drivers, and blue points are cancer-associated genes.

Overall, each centrality measure exhibits a similar distribution across the three CCNs, but the positions of the red and blue points vary. The CHR CCN has only five impacting genes, making it difficult to identify clear patterns. In this network, drivers and cancer-associated genes intermingle, occupying medium to high ranges in Degree, Closeness, and Betweenness. In the DNA CCN, with 13 impacting genes, the red and blue points are more evenly distributed in the middle, showing no clear distinction between drivers and cancer-associated genes, and they do not appear at the distribution extremes. Conversely, in the PCD CCN, drivers tend to occupy the top values in Degree, Closeness, and Betweenness, with low Clustering values. Additionally, there is a noticeable separation where known drivers tend to lead in these centrality measures, followed by cancer-associated genes.

Figure 4 shows that no single centrality measure is sufficient to characterise the genes impacting $\beta _2$ structures. Although traditional centrality measures focus on nodes and edges within the network, they fail to capture the complexity of high-dimensional structures associated with these genes. This indicates that understanding the role of these genes requires going beyond basic centrality measures to account for the more complex, high-dimensional interactions and structures present in the network.

### Enrichment analysis of impactful genes

To expand the biological role of the impactful genes, we performed functional enrichment analyses using the online tools KEGG^58–60^, DAVID^61^, and DGIdb^62^.

Using KEGG, we focused on the *Pathways in Cancer* module, analyzing the 35 genes listed in Tables 1 and 2. Of these, 16 genes were mapped to the *KEGG Pathways in Cancer*, consisting of 11 known driver genes and 5 cancer-related genes. Figure 5 in the supplementary material shows the pathway map, highlighting which genes match with pathway their the specific locations.

With DAVID, we identified several enriched biological associations, here we focus on *Functional*_*Annotations*, specifically the *UP*_*KW*_*BIOLOGICAL*_*PROCESS* (UP_KW stands for UniProt Keywords) . Table 3 shows the biological processes, the number of impactful genes involved, and the associated p-value. The processes of Apoptosis, DNA Repair, DNA Damage, and DNA Recombination are highly associated with cancer and match the SPNs we used to create the CCNs.

**Table 3:** Impactful genes participating in biological processes.

Term	Count	P-value
Apoptosis	14	< 0.01
DNA damage	13	< 0.01
DNA repair	12	< 0.01
Host–virus interaction	11	< 0.01
DNA recombination	3	< 0.05
Neurogenesis	4	< 0.05
Necrosis	2	< 0.05

**Figure 5:**
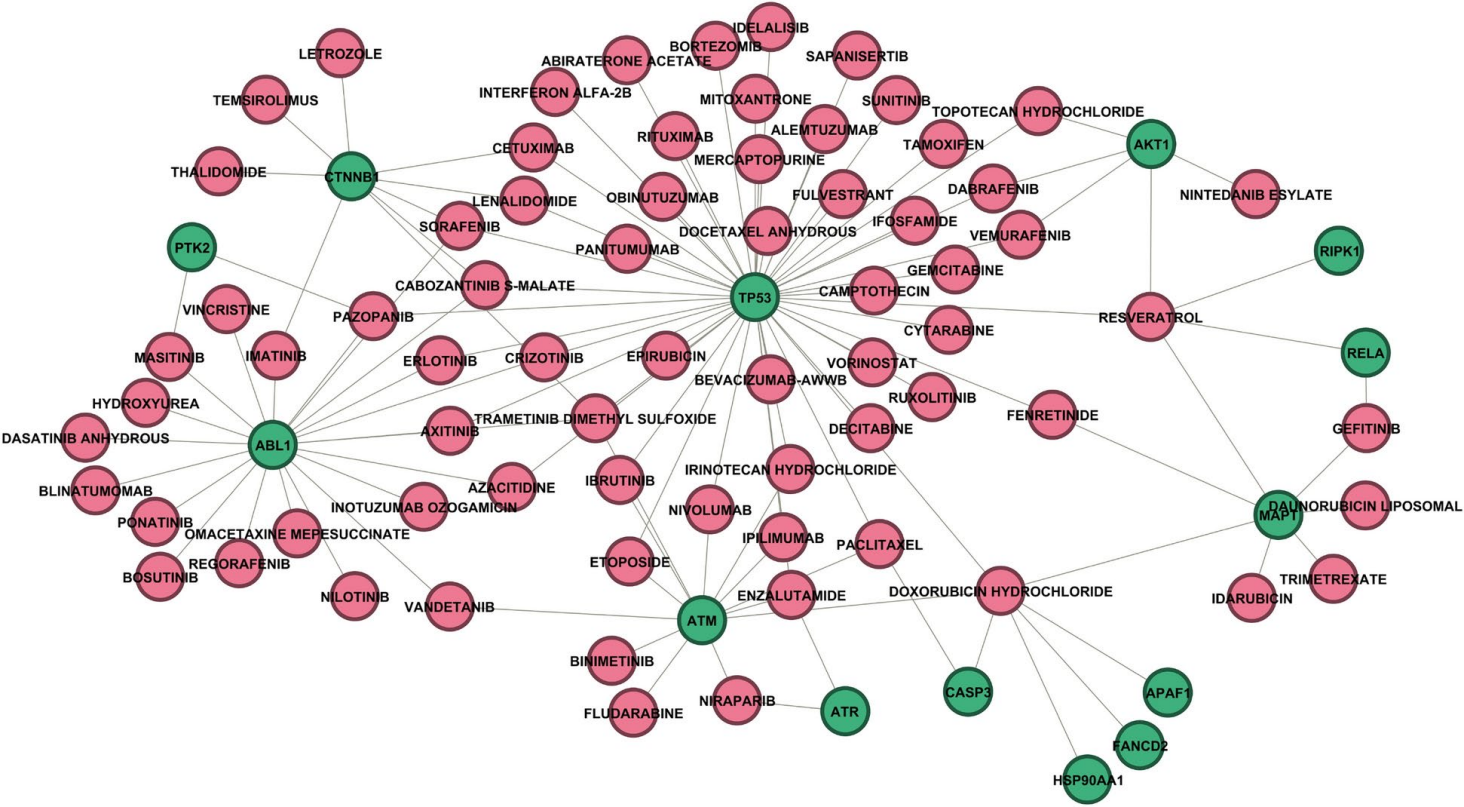
Gene drug interaction network.

Finally, using DGIdb, we investigated the association of impactful genes with drugs. A total of 1,857 interactions were identified. After filtering for interactions involving FDA-approved drugs and limiting only those with antineoplastic activity, we found 114 interactions. A complete table detailing all these interactions is available in the supplementary material. Figure 5 shows the interactions as a bipartite network, presenting only the largest connected component. Green nodes are genes, and red nodes are drugs.

This multi-faceted approach highlights the functional significance and potential clinical relevance of the impactful genes, offering insights into their roles in cancer biology and therapeutic applications.

### Other methods exploring high-order structures in cancer subnetworks

High-order structures extracted from PPINs have been used in Graph Neural Networks (GNNs)-based methods to identify cancer genes. Methods like EMOGI^63^ and CGMega^64^ integrate multi-omics data with PPINs to analyze gene interactions in high-dimensional structures. The high-order structures in these approaches refer to modules derived from PPINs, which are created based on biological and topological features, often linked by functional relationships or shared characteristics. For instance, CGMega identifies a core subnetwork of key pairwise relationships for cancer gene prediction and uses 15-dimensional importance scores to assess the contribution of each gene (i.e. node). Similarly, EMOGI enriches genes with multi-omic and topological features extracted from PPINs, clusters genes based on feature contributions, and identifies 45 modules, with the largest (149 genes) forming the core subnetwork for cancer gene classification.

Our method differs from GNNs-based methods by employing PH to analyze the topological structures of CCNs. PH, rooted in algebraic topology, focuses on the distance between nodes to build simplexes and identify high-order structures that persist across time. This approach reveals complex topological features, such as the impact of cancer genes on $\beta _2$ structures, highlighting how genes contribute to maintaining the overall topology of the network. Unlike GNNs, PH offers a unique perspective by capturing topological features in increasing dimensions, revealing gene relationships beyond simple pairwise interactions.

By combining GNNs’ predictive capabilities with PH’s structural insights, researchers can develop a comprehensive framework for studying cancer networks. This integration can improve the identification of driver genes and enhance the understanding of their roles in the complex biological processes underlying cancer.

## Conclusion

The study presents a novel approach to identifying known drivers and cancer-associated genes within cancer networks extracted from pathways using Persistent Homology. We constructed Cancer Consensus Networks by integrating mutation data from six types of cancer and three main biological functions. We measure the impact of removal of each gene in cancer networks with respect to its role in the construction of higher-order structures. We complement the analysis using centrality measures to verify if traditional measures can capture the impacting genes. The results demonstrate that only a few genes decrease the number of voids ($\beta _2$ structures). In particular, all impactful genes are established cancer drivers or cancer-associated genes, supported by existing literature, with the potential to be new drivers. We also perform functional enrichment analysis on the impactful genes, showing their association with cancer pathways, biological functions and relationship with antineoplastic drugs. Although not every driver or cancer-associated gene impacts $\beta _2$, no passenger gene does. The pipeline used in this work demonstrated high precision and low to average recall in distinguishing drivers from passengers. Although centrality measures alone do not fully characterise drivers and cancer-associated genes in CCNs, these genes generally exhibit low clustering and medium to high degree, closeness, and betweenness centrality values. This centrality profile, combined with the observation that no passenger mutations impact higher-order structures, can be utilized to evaluate candidate driver genes. Their topological characteristics can help determine their biological function as drivers or passengers.

## Supplementary Information

Supplementary Information 1.

Supplementary Information 2.

## Data Availability

The mutation datasets are from a TCGA study^35^ and can be downloaded from cBioPortal. All code, input, and output files are on GitHub: https://github.com/RodrigoHenriqueRamos/Identifying-Key-Genes-in-Cancer-Networks-Using-Persistent-Homology
