# Supplementary material for: Identifying key genes in cancer networks using persistent homology
Source: Sci Rep. 2025 Jan 22;15:2751. doi: 10.1038/s41598-025-87265-4 (PMC11751331; doi:10.1038/s41598-025-87265-4)
Supplement: Supplementary file 2 — Supplementary Information 2. [file 41598_2025_87265_MOESM2_ESM.pdf]

Supplementary Material

SM 1 - Simplicial Complex and Filtration

A simplicial complex is a collection of simplices $\sigma = [0,...,k]$ with dimension k. A 0-simplex corresponds to a vertex $[0]$, a 1-simplex to an edge $[0,1]$, a 2-simplex to a triangle $[0,1,2]$, a 3-simplex to a tetrahedron $[0,1,2,3]$, and so forth. Figure 1-(A) provides a visual representation of simplices. In a simplicial complex K, if a simplex $\sigma \subset K$, then every non-empty subset $\tau \subset \sigma$ is also a part of K. Additionally, two k-simplices in K are either disjoint or intersect in a lower-dimensional simplex that is also contained within K.

To analyze the persistent homology of a dataset, a sequence of simplicial complexes $K_1 \subseteq K_2 \subseteq ... \subseteq K_n$, known as a filtration, must be constructed. A common filtration is the Vietoris-Rips complex[1], which constructs a topological space from a metric space using a distance r. By selecting a parameter $r > 0$, we construct a simplicial complex K_r, where a k-simplex is included in K_r if the distance between any two points forming the simplex is less than $2r$. It is important to note that if $r_2 \geq r_1$, then $K_{r1} \subseteq K_{r2}$. Figure 1-(B) exemplify a filtration over 4 points, and the birth and death of topological structures.

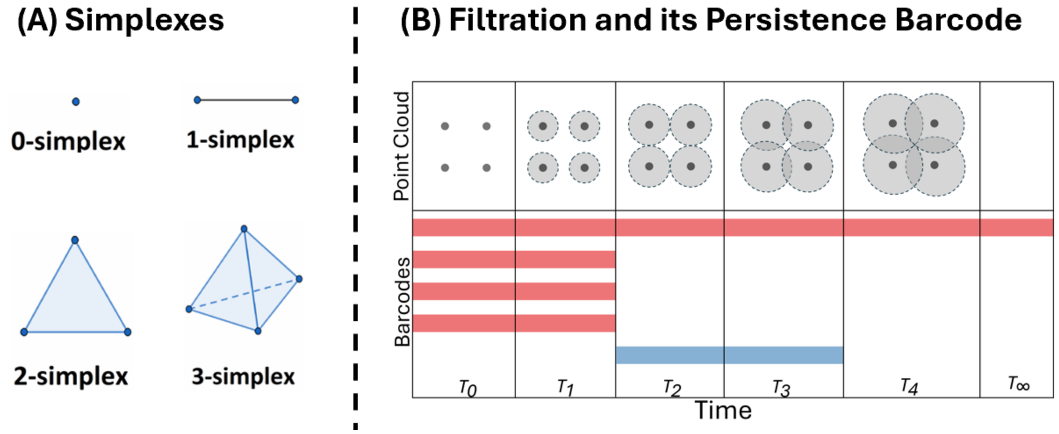

Figure 1. (A) Shows examples of simplexes of dimensions 0, 1, 2 and 3. (B) Presents the Vietoris-Rips filtration for a point cloud consisting of four equidistant points and the Persistent Barcode capturing the birth and death of topological structures.

SM 2 - Chains and Boundaries

To describe the topological properties in a simplicial complex K_r, we must utilize concepts from algebraic topology. In a simplicial complex K, for $k \geq 0$, a k-chain C_k is defined as a vector space whose basis consists of a set of k-simplices in K. The dimension of C_k is determined by the number of elements in this basis. These vector spaces are composed of all linear combinations $c = \sum_i a_i \sigma_i$, where $a_i \in \mathbb{Z}p$ (p being a prime integer) and the summation runs over all k-simplices σ_i in K. Within this framework, the linear transformation $\partial_k : C_k \rightarrow Ck - 1$ can be introduced. This map, known as the boundary map, is defined as $\partial_k([0,...,k]) = \sum_{i=0}^{k}(-1)^i[0,...,\hat{\imath},...,k]$, where the notation $\hat{\imath}$ indicates the removal of that vertex. The boundary map applied to a simplex yields the alternating sum of the simplices along the boundary. Recall that the boundary of a k-simplex σ is the union of the (k - 1)-subsimplices $\tau \subseteq \sigma$. Additionally, note that the composition $\partial_k \; o \; \partial_{k+1} = 0$. This implies that $im(\partial_{k+1}) \subseteq ker(\partial_k)$.

Consider a chain complex $... \rightarrow C_{k+1} \xrightarrow{\partial_{k+1}} C_k \xrightarrow{\partial_k} C_{k-1} \rightarrow ... \rightarrow C_2 \xrightarrow{\partial_2} C_1 \xrightarrow{\partial_1} C_0 \xrightarrow{\partial_0} 0$, and define two subspaces of C_k using the kernel and image of the boundary maps, namely $Z_k = ker(\partial_k)$ (k-cycles) and $B_k = im(\partial_{k+1})$ (k-boundaries). It is important to note that B_k is a subspace of Z_k, and thus we can define $H_k = Z_k/B_k$, which represents the quotient of these vector spaces. The kth Betti number[1], denoted by β_k, is defined as $\beta_k = dim(H_k) = dim(Z_k) - dim(B_k)$. Betti numbers quantify the number of "holes" in a simplicial complex K. Specifically, β_0 indicates the number of connected components in K, β_1 the number of cycles in K, β_2 the number of 2-dimensional holes, and more generally, β_k represents the number of k-dimensional holes in K. Betti numbers are a topological invariant, meaning that topologically equivalent spaces share the same Betti numbers. Figure 2 provides an example of calculating the Betti number β_1 for two distinct simplicial complexes.

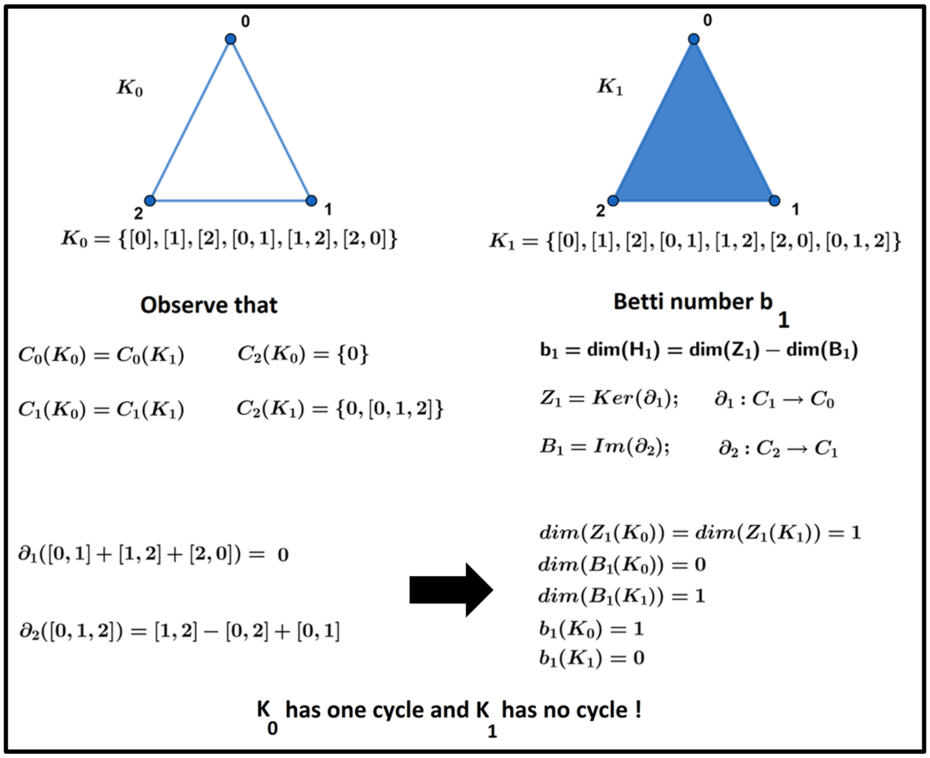

Figure 2. The Betti number β_1 for the complexes K_0 and K_1. Observe that the only element in C_1 that lies within the kernel of ∂_1 is $[0,1]+[1,2]+[2,0]$, leading to $dim(Z_1(K_0)) = dim(Z_1(K_1)) = 1$. Additionally, note that the boundary of the simplex $[0,1,2]$ forms the oriented triangle $[1,2]-[0,2]+[0,1]$. As a result, $dim(B_1(K_0)) = 0$, $dim(B_1(K_1)) = 1$, yielding $b_1(K_0) = 1$ and $b_1(K_1) = 0$. This indicates that K_0 contains a cycle, whereas K_1 does not.

SM 3 - Calculating PH in Python

Listing 1 implements part of Algorithm 3 in Python: the functions $fromNetworkToPH$ and $getOnlyB2$. In Algorithm 3, these functions are abstracted to convey the general idea behind calculating the impact. In Listing 1, the $fromNetworkToPH$ function is implemented from line 7 to line 20, while lines 22 to 24 demonstrate how it is possible to extract the number of structures per dimension, demonstrating the idea behind $getOnlyB2$.

A fundamental step in calculating PH is defining a metric space to calculate distances. Since we use networks, we create a distance matrix by calculating the shortest path from all vertices to all vertices (line 10). This matrix must be symmetric (lines 12 and 13) for the correct calculation of PH.

Line 16 is discussed in the next section. Line 19 defines the maximum dimension in which the PH will be calculated. $dim = 1$ for connected components (β_0), $dim = 2$ for cycles (β_1), $dim = 3$ for voids (β_2), and so on. Line 20 effectively computes persistent homology, a complex process (see previous sections of the supplementary material) that the GUDHI library abstracts. The computational cost in time and space for computing Vietoris-Rips is exponential, raising the number of points N to the value defined in dim:

$$\sum_{i=1}^{\text{dim}} N^i = N^{\text{dim}} + N^{\text{dim}-1} + \cdots + N^1$$

In our case, N is the number of vertices in the network, 170, and dim is 3.

Listing 1. Python script for analyzing a CCN with Vietoris-Rips Complex

```
1  import networkx as nx
2  import pandas as pd
3  import gudhi as gd
4  import matplotlib.pyplot as plt
5
6  # Open a CCN
7  PCD_CCN = nx.read_edgelist('1 output\Consensus PCD')
8
9  # Create a Distance Matrix (dm), the space metric to calculate PH
10 dm = pd.DataFrame(dict(nx.shortest_path_length(PCD_CCN)))
11 # Make sure the dm is symmetrical
12 dm.sort_index(inplace=True)
13 dm = dm[sorted(dm.columns)]
14
15 # Create an object to work with the Vietoris-Rips Complex
16 rips_complex = gd.RipsComplex(distance_matrix = dm.values)
17
18 # Set max dimension and calculate PH
19 dim = 3
20 barCode = rips_complex.create_simplex_tree(max_dimension = dim).persistence()
21
22 print("Number of Betti 0 Structures:", len([b for b in barCode if b[0] == 0]))
23 print("Number of Betti 1 Structures:", len([b for b in barCode if b[0] == 1]))
24 print("Number of Betti 2 Structures:", len([b for b in barCode if b[0] == 2]))
```

The output for Listing 1 is:
Number of Betti 0 Structures: 170
Number of Betti 1 Structures: 23
Number of Betti 2 Structures: 10

SM 4 - Impact of the r parameter on the Vietoris-Rips Complex calculation

The previous section indicates that the computational complexity of Vietoris-Rips is exponential. This is true if we let the filtering process run until the radius around each point covers all other points (more details in Figure 1 of this supplementary material). This way, all possible simplices up to the chosen dimension are created. However, it is possible to limit the size of the radius r around each point, allowing only points with distance <= r to construct simplices. This process effectively reduces the computational cost but at the cost of potentially not finding all existing structures.

Listing 2 exemplifies this phenomenon in a sample network. In lines 8 and 9, the value of r is limited to 1 and 2. In line 11, the parameter is omitted and the filtration runs by default. In line 13, we set the dimension to 5 in order to extract more

structures and visualize the impact of r. The second part of the figure presents the network plot and barcodes for different values of r. All edges have weight 1 in the network, so when limiting $r = 1$, only 2-simplex (edges) are found during the filtration. Thus, the filtration ends when all nodes are connected to their neighbors, but before finding any cycle. When limiting $r = 2$, several cycles are found, most dying at time 2. However, three cycles persist beyond time 2. Since these cycles have no death time, they are treated as infinite. The last subplot shows the complete filtration up to dimension 5. One of the cycles treated as infinite dies at time 3, and the other two die at time 4. Furthermore, we also observe that the complete filtration captures two structures of dimension 3 and three structures of dimension 5. The network used as an example (a Tutte Graph) has 46 vertices, and we calculate the PH up to the fifth dimension. Limiting $r = 1$, the persistence calculation occurs in less than a nanosecond, limiting $r = 2$ takes 1 microsecond, and without limitation, it takes 15.5 seconds.

Listing 2. Python script for Rips complex construction with different maximum edge lengths

```python
tutteGraph = nx.generators.tutte_graph()
dm = pd.DataFrame(dict(nx.shortest_path_length(tutteGraph)))
dm.sort_index(inplace=True)
dm = dm[sorted(dm.columns)]

# To construct the Rips complex, a ball of radius r is grown around each point.
# We can limit r using the max_edge_length parameter.
rips_complex_r1 = gd.RipsComplex(distance_matrix = dm.values, max_edge_length=1)
rips_complex_r2 = gd.RipsComplex(distance_matrix = dm.values, max_edge_length=2)
# If omitted, the r grows until it encompasses all points.
rips_complex_default = gd.RipsComplex(distance_matrix = dm.values)

dim = 5
BarCode_r1 = rips_complex_r1.create_simplex_tree(max_dimension = dim).persistence()
BarCode_r2 = rips_complex_r2.create_simplex_tree(max_dimension = dim).persistence()
BarCode_default = rips_complex_default.create_simplex_tree(max_dimension = dim).persistence()
```

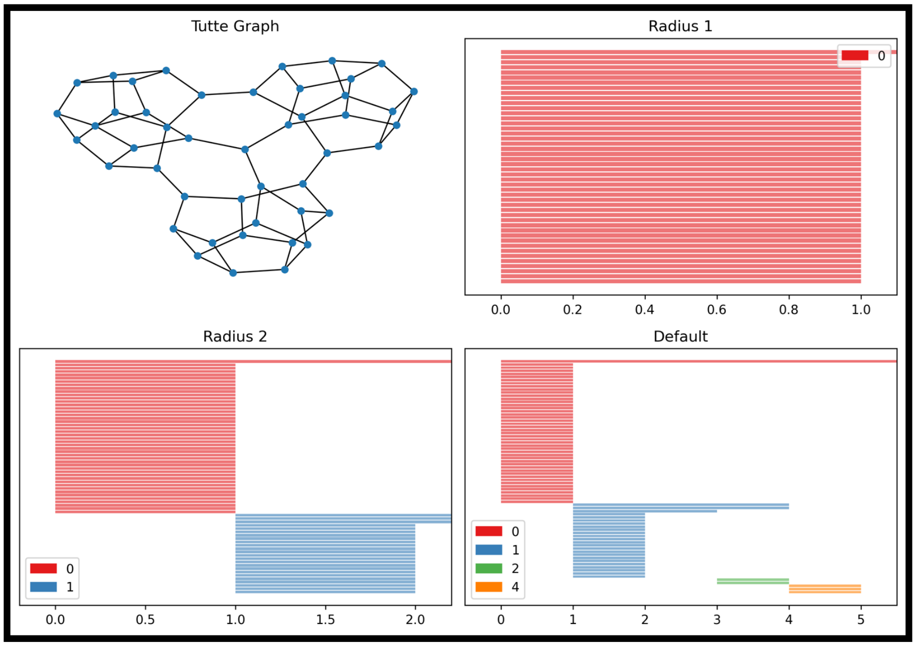

Figure 3. Impact of limiting r.

In the case of the CCNs, the filtration was performed without limiting r, which impacts the computational cost but ensures that all structures are found. All high-dimensional structures are born at time 1 and die at time 2, as exemplified by Figure 4. Note that the only continuous bar is a β_0. This is why our analyses consider the number of structures and not their persistence.

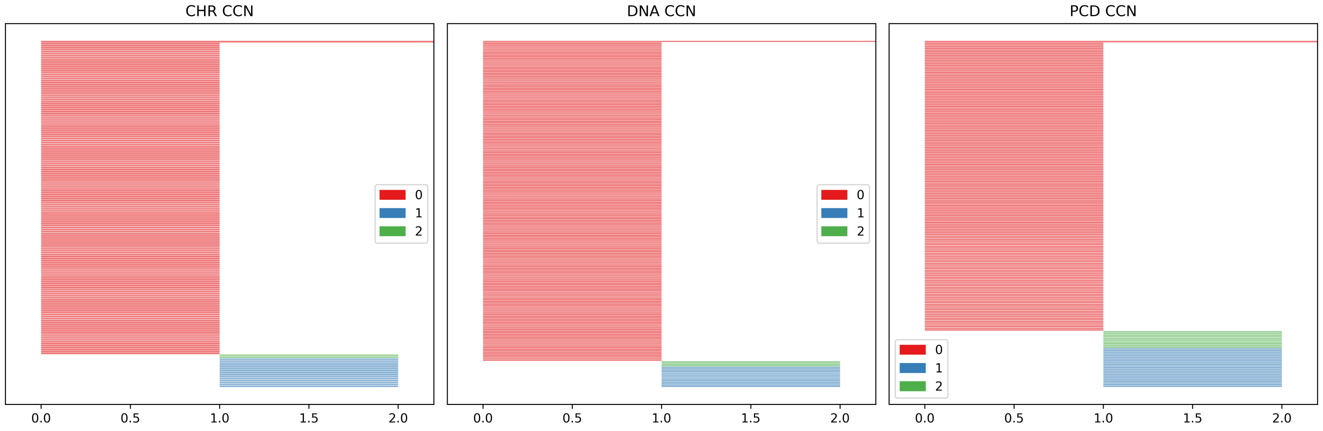

Figure 4. Barcodes for CCNs.

SM 5 - Association of Genes Affecting β_2 Structure to Known Cancer Mechanisms

We used the KEGG database, specifically the Pathways in Cancer: https://www.genome.jp/pathway/hsa05200, to analyze the 35 genes that impact β_2. 16 genes were identified, 11 known drivers (ABL1, AKT1, CASP3, CASP8, CTNNB1, EP300, HSP90AA1, PTK2, RELA, STAT3, TP53), and 5 cancer-associated genes (APAF1, BAD, BIRC2, RAD51,ROCK1). Figure X shows the KEGG's Pathways in Cancer, highlighting impactful genes in pink.

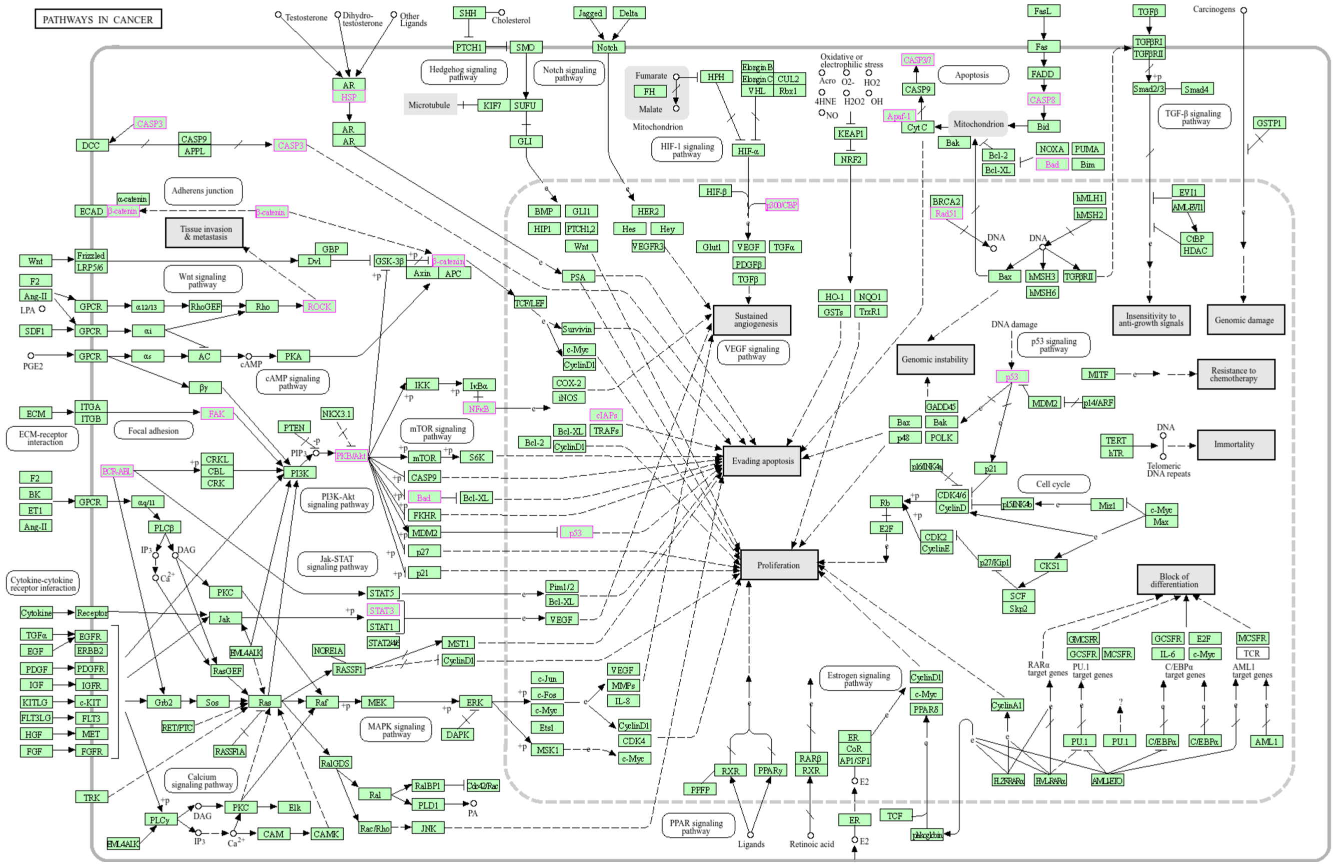

Figure 5. KEGG[2–4] Pathways in Cancer Enriched with Impactful Genes.

References

1. Chazal, F. & Michel, B. An introduction to topological data analysis: fundamental and practical aspects for data scientists. *arXiv preprint arXiv:1710.04019* (2017).

2. Kanehisa, M. & Goto, S. Kegg: kyoto encyclopedia of genes and genomes. *Nucleic acids research* **28**, 27–30 (2000).

3. Kanehisa, M. Toward understanding the origin and evolution of cellular organisms. *Protein Sci.* **28**, 1947–1951 (2019).

4. Kanehisa, M., Furumichi, M., Sato, Y., Kawashima, M. & Ishiguro-Watanabe, M. Kegg for taxonomy-based analysis of pathways and genomes. *Nucleic acids research* **51**, D587–D592 (2023).
